# Supplementary material for: DIOPT: the DRSC Integrative Ortholog Prediction Tool, 2026 update
Source: bioRxiv. 2026 Apr 16:2026.04.15.718708. Preprint. [Version 1] doi: 10.64898/2026.04.15.718708 (PMC13104911; doi:10.64898/2026.04.15.718708)

**Sup Figure 1. Metrics used to set the cutoff of robustness score for FlyOrthoList**


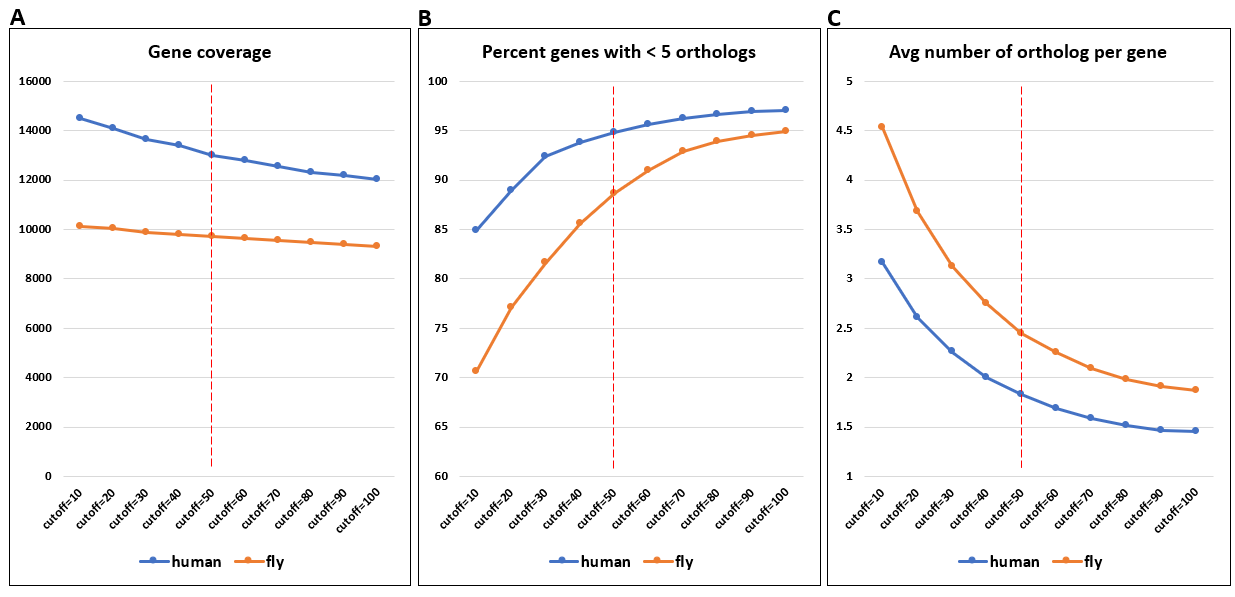


**Sup Figure 2. DIOPT supports ortholog mapping at many public resources.** Either DIOPT database exports or results from the customizable DIOPT pipeline have been integrated into the indicated public resources.


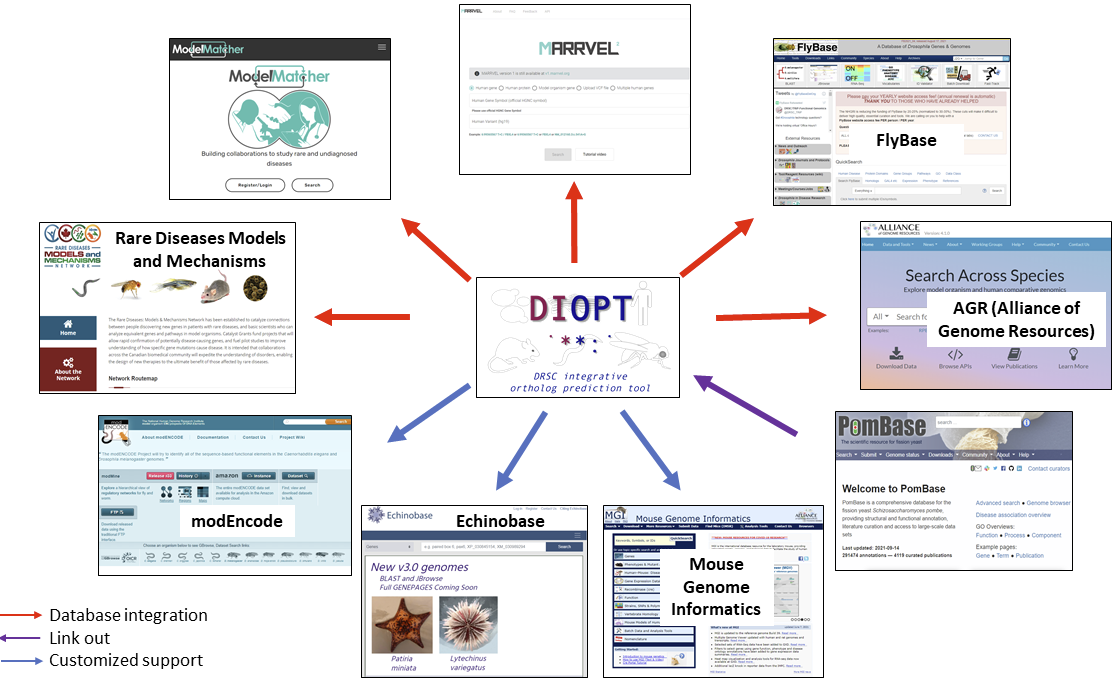

Supplement: Supplement 4 [file media-4.docx]
